# Advances in Real-Time MRI–Guided Electrophysiology

**DOI:** 10.1007/s12410-019-9481-9

**Published:** 2019-02-12

**Authors:** Rahul K. Mukherjee, Henry Chubb, Sébastien Roujol, Reza Razavi, Mark D. O’Neill

**Affiliations:** 1 School of Biomedical Engineering and Imaging Sciences, King’s College London, 4th Floor, North Wing, St Thomas’ Hospital, London, SE1 7EH UK; 2 Department of Cardiology, King’s College Hospital NHS Foundation Trust, London, UK

**Keywords:** Magnetic resonance imaging, Electrophysiology, Real-time, Atrial flutter, Ventricular tachycardia, Ablation

## Abstract

**Purpose of Review:**

Theoretical benefits of real-time MRI guidance over conventional electrophysiology include contemporaneous 3D substrate assessment and accurate intra-procedural guidance and evaluation of ablation lesions. We review the unique challenges inherent to MRI-guided electrophysiology and how to translate the potential benefits in the treatment of cardiac arrhythmias.

**Recent Findings:**

Over the last 5 years, there has been substantial progress, initially in animal models and more recently in clinical studies, to establish methods and develop workflows within the MR environment that resemble those of conventional electrophysiology laboratories. Real-time MRI-guided systems have been used to perform electroanatomic mapping and ablation in patients with atrial flutter, and there is interest in developing the technology to tackle more complex arrhythmias including atrial fibrillation and ventricular tachycardia.

**Summary:**

Mainstream adoption of real-time MRI-guided electrophysiology will require demonstration of clinical benefit and will be aided by increased availability of devices suitable for use in the MRI environment.

## Introduction

Electroanatomic mapping (EAM) systems are commonly used in the conventional electrophysiology laboratory for the guidance of complex arrhythmia procedures. EAM can be used to generate 3D chamber geometry, enable non-fluoroscopic localisation of catheters, delineate areas of electrical substrate and record location of ablation lesions [1]. There are several limitations however which may account for the modest long-term success rates of ablation targeting EAM-defined arrhythmia substrate in the setting of complex arrhythmias [2, 3]. Bipolar voltage mapping typically has a limited field of view and may miss intramural or epicardial substrate, whereas the use of magnetic resonance imaging (MRI) can identify candidate arrhythmogenic substrate in 3D [4]. Voltage amplitude is also affected by many factors other than the histological characteristics of the underlying tissue including recording electrode size, inter-electrode distance, direction of activation wavefront, catheter orientation and force of contact [5, 6]. Catheter location in conventional EAM systems is triangulated using magnetic-based sensing or impedance-based tracking and displayed on approximate geometries of cardiac chambers [1]. Visualisation of soft tissue is limited and requires additional imaging equipment in the conventional electrophysiology laboratory (e.g. intra-cardiac ultrasound) whilst estimation of ablation lesion dimensions is dependent on mathematical modelling of limited biophysical parameters (time, power, force, change in impedance) rather than a direct visualisation of transmural, durable lesion formation [7].

Conversely, MRI could offer a number of benefits to the electrophysiologist including 3D evaluation of chamber geometry and myocardial structural substrate to complement functional electrophysiological assessment, accurate tracking of catheters to improve intra-procedural guidance and assess ablation lesion formation with soft tissue visualisation [8]. Although centres have integrated anatomical and scar data from CT or MRI into the navigation system during catheter ablation [9•, 10], these techniques invariably result in registration errors of up to 3.5 mm [11]. Additionally, conformational changes within cardiac chambers as a result of different loading conditions between the time of imaging and intervention as well as translational changes due to patient movement, cardiac and respiratory motion could all lead to discrepancies between structural data and electrical substrate with potential consequences for the efficacy of catheter ablation [12]. The mean maximum amplitude of cardiac and respiratory motion, for example, during EAM has been estimated to be in the region of 10.2 ± 2.7 mm and 8.8 ± 2.3 mm respectively [13].

**Fig. 1:**
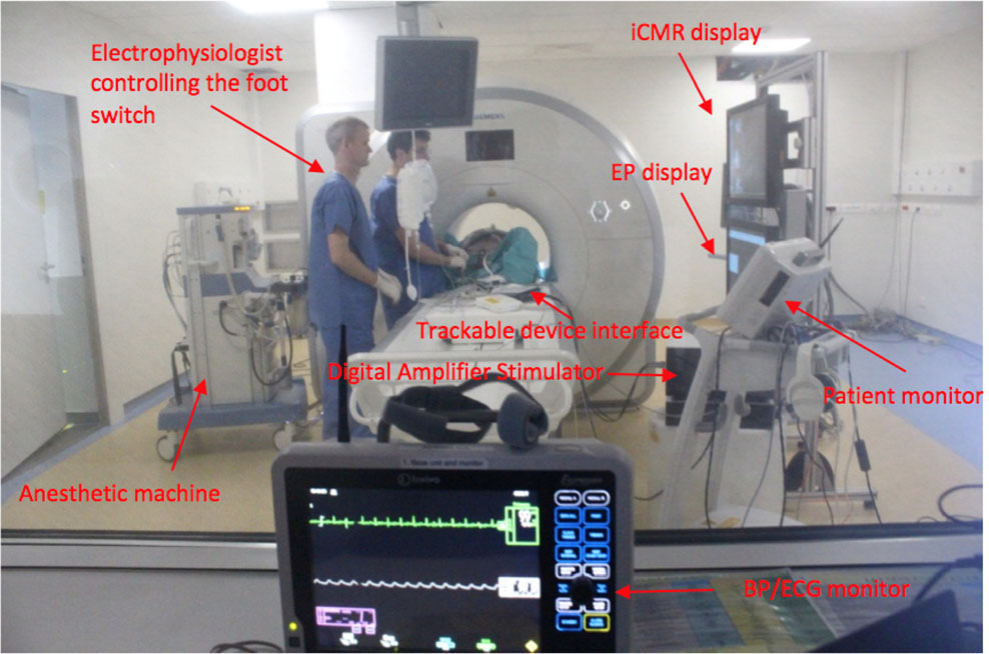
Interventional MRI (iCMR) set-up to perform electrophysiology (EP) procedures. An example set-up for an animal (porcine) study is shown here. The electrophysiologist can view both electrical and imaging data on dedicated displays. A foot switch can be used to start/stop MRI scanning in the same way as during fluoroscopy-guided procedures. Subject monitoring (Invivo, Gainesville, Florida): four-lead surface ECG, arterial blood pressure monitoring and oxygen saturation assessment are available and can be viewed both inside the scanner room and control room. MR-compatible electrophysiology catheters are connected to a tracking interface which can transmit tracking data to the image guidance platform. Radiofrequency (RF) generator, irrigation pump and EP recording system are located within the control room

Real-time MRI-guided electrophysiology offers a potential solution to overcome the limitation of image integration as well as utilise the benefits of MR-based catheter tracking and lesion assessment that can only be performed inside a MRI scanner [8]. Over the last decade, development of MR-conditional devices and imaging techniques has led to the application of interventional MRI in the setting of cardiac electrophysiology (Fig. 1) but a number of challenges have been identified. These include the recording of high-quality electrograms in an environment with significant electromagnetic interference, the development of clinical-grade MR-compatible devices with similar physical capabilities as their conventional counterparts and establishing robust tools for real-time lesion assessment [12]. On a practical level, effective communication is difficult between team members in a noisy environment and novel workflows are evolving to accommodate this hurdle. In this article, we review the progress made within real-time MRI-guided electrophysiology, highlighting the unique challenges we have encountered and their solutions as well as evaluating its application in the treatment of patients with cardiac arrhythmias.

## Electrogram Fidelity in the MRI Environment

Accurate recording and characterisation of both surface electrocardiogram (ECG) and intra-cardiac electrograms (EGM) are an essential component of cardiac electrophysiology procedures. During catheter ablation of simple arrhythmias such as atrial flutter, demonstration of bidirectional conduction block created at the cavo-tricuspid isthmus and proven by sequential, timed EGM recordings made along the ablation line during coronary sinus pacing is necessary [14]. For complex arrhythmias such as ventricular tachycardia, substrate-based mapping techniques rely on the correct identification of low voltage regions, annotation of abnormal potentials and robust characterisation of electrogram morphologies [5].

**Fig. 2:**
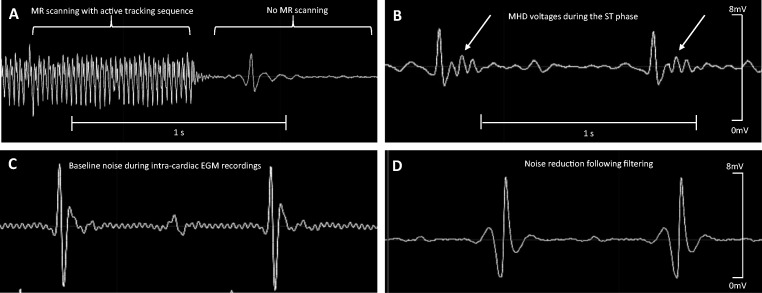
Recording surface ECG and intra-cardiac EGMs inside a MRI scanner. Significant distortions inside a MRI scanner can occur on both the surface ECG and during intra-cardiac EGM acquisition due to (1) time-varying MR gradient fields, (2) RF pulses during MR scanning that couple to conductive material (**a**) and (3) magneto-hydrodynamic (MHD) voltages due to the static magnetic field (**b**). Noise filtering technologies are required to improve intra-cardiac EGM fidelity and reduce baseline noise (**c**, **d**)

The MRI environment is considered relatively ‘hostile’ for the rigorous recording of EGMs as there are many sources of artefact that may distort signals. During MR scanning, RF pulses are deployed that are coupled with fast-switching magnetic gradients which can induce electromagnetic fields around the subject and create voltage artefacts on both the surface ECG and intra-cardiac EGMs [15]. A second source of signal distortion is the magneto-hydrodynamic effect (MHD). The static magnetic field (*B*_0_) can lead to MHD voltages due to the flow of electrically charged blood particles through the aortic arch in a direction perpendicular to *B*_0_. MHD voltages are superimposed primarily during the S-T phase of the cardiac cycle (period of peak flow) and can have a similar frequency spectrum and magnitude to real electrogram signals (Fig. 2) [16]. The size of MHD voltages may also be affected by the heart rate as a different volume of blood may be ejected from the left ventricle—this may be relevant during EGM recordings in the context of tachycardia or pacing. A third source of artefacts are time-varying magnetic fields that provide position-dependent variation in MR field strength but can lead to induced electric currents in conducting tissues as well as connecting wires [17].

Following the application of a low-pass filter (300 Hz), high-pass filter (30 Hz), notch filter (60 Hz) and RF filters to reduce the 64 MHz signal from the MRI scanner, Nazarian et al. [18] demonstrated the ability to record both atrial and ventricular potentials in normal canines and healthy patients including distinguishing small His potentials with good fidelity. Subsequent studies in patients with atrial flutter have shown that double potentials can be detected along and conduction detour around an ablation line [19••, 20••]. An experimental study in an ovine model estimated that the peak-to-peak amplitude of baseline noise inside the MRI scanner (without scanning) is 10-fold higher than in a conventional X-ray fluoroscopy laboratory (0.10 vs 0.01 mV) whilst the signal-to-noise ratio (SNR) may vary depending on the MRI sequence used during scanning [21•]. In this study, a low-pass filter with a cut-off frequency of 120 Hz gave the best quality EGM signals compared to filter cut-offs at 240 and 500 Hz [21•]. It remains unclear, however, whether low-amplitude abnormal potentials such as fractionated EGMs or late potentials with an amplitude < 0.1 mV can be detected robustly inside a MRI scanner with limited evidence available from one study [22]. Although a lower value low-pass filter may give the best quality normal EGM signals, it may risk losing the fine high-frequency components of abnormal EGMs that are important in substrate evaluation. Further developments in signal processing technologies are needed to improve EGM fidelity, and there has been interest in the application of techniques such as adaptive noise cancellation [23] artefact modelling and Bayesian filtering for this purpose [15].

## Passive and Active Catheter Tracking

**Fig. 3:**
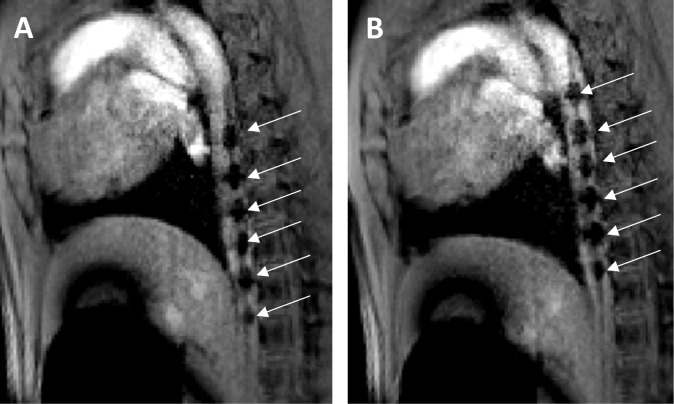
Passive catheter tracking with negative contrast using a spoiled gradient echo sequence in coronal orientation with a water-selective excitation pulse as a catheter is advanced through the aorta (**a**, **b**). Arrows show location of electrodes on a hexa-polar catheter

There are two principle methods of intra-procedural guidance during MRI-guided electrophysiology—active and passive catheter tracking. Passive catheter tracking enables either positive or negative contrast visualisation by integration of susceptibility artefacts generated from ferromagnetic/paramagnetic materials embedded within a catheter [24] or using gadolinium or CO_2_/N_2_O-filled balloon catheters [25]. Negative contrast techniques may be associated with signal void artefacts, but these may be discarded through segmentation of catheter markers—Fig. 3. Typically, during passive tracking an in-plane resolution of around 1 mm, slice thickness of 6 mm and temporal resolution of 4 frames/s has been reported [26]. Tip tracking accuracy using gadolinium-filled balloon catheters has been estimated to be in the region of ± 0.41 mm with imaging reconstructions displayed at a frame rate of around 3 frames/s [27]. Recently, application of a positive-contrast based passive tracking sequence using partial saturation magnetisation preparation has been shown to provide improved simultaneous visualisation of both anatomy as well as gadolinium-filled catheters [28]. However, susceptibility artefacts during passive tracking can make devices with ferromagnetic/paramagnetic materials appear larger than their actual size as well as potentially obscure surrounding tissue. Furthermore, with all passive tracking techniques, if the device moves out of the imaging plane, the operator is required to relocate the device which can be time-consuming [29]. It is also more difficult to track catheters within the expansive 3D space of cardiac chambers compared to in-plane tracking within the lumen of a vessel (e.g. aorta).

**Fig. 4:**
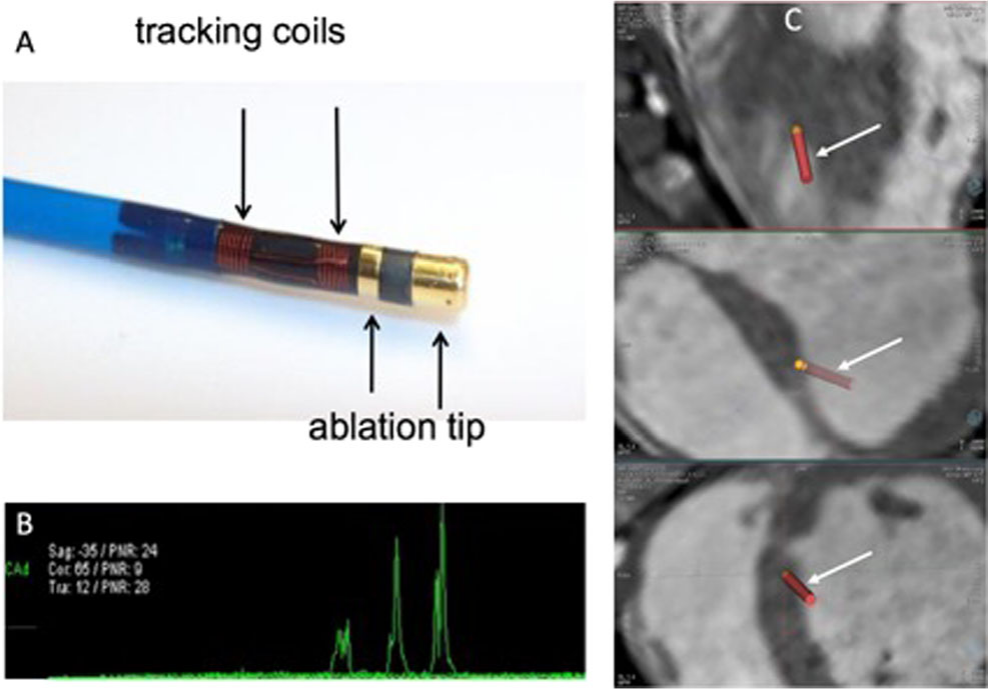
Active catheter tracking using a MR-compatible catheter with gold-tip electrodes and two 2.5-mm solenoid receive coils (**a**). Using a dedicated tracking sequence, the *X*, *Y* and *Z* coordinates of the catheter micro-coils can be determined in 3D space (**b**) whilst the catheter tip can be displayed as a red icon in three orthogonal MRI projections to guide a MR-EP procedure (**c**)

Active catheter tracking is an alternative technique whereby micro-coils embedded within the catheter produce a receiver signal to determine location of the device (Fig. 4). An active tracking sequence consisting of intermittent non-selective excitations combined with spatially encoding gradients enables measurement of 1D projections in each space dimension and estimation of the 3D coordinates of the position of the micro-coils [20••, 30]. Real-time estimates of the position of micro-coils can be used to estimate catheter tip orientation. This information can also be used for real-time slice tracking to automatically maintain the catheter tip in the imaging plane during navigation [20••]. The precision of active tracking during ex vivo technical validation experiments has been estimated to be in the region of 0.90 ± 0.58 mm along the axis of the catheter whilst the angular deviation of catheter orientation from its true direction was 8.5^0^ ± 3.6^0^ [20••]. Active tracking has been used to perform coronary sinus intubation, activation mapping and trans-septal left atrial access in swine [31] as well as to guide cavo-tricuspid isthmus ablation in patients with atrial flutter [19••, 20••]. Furthermore, active tracking has also enabled epicardial electroanatomical mapping and ablation in the porcine left ventricle inside the MRI scanner [32]. Data comparing a conventional EAM system to EAM using active tracking inside a MRI scanner are scarce [33] and are confined to normal porcine hearts. Further head-to-head comparisons between active tracking and conventional magnetic-sensor based or impedance-based tracking to guide catheters to target locations are required to determine the spatial accuracy of the techniques.

## Image Guidance Platforms

**Fig. 5:**
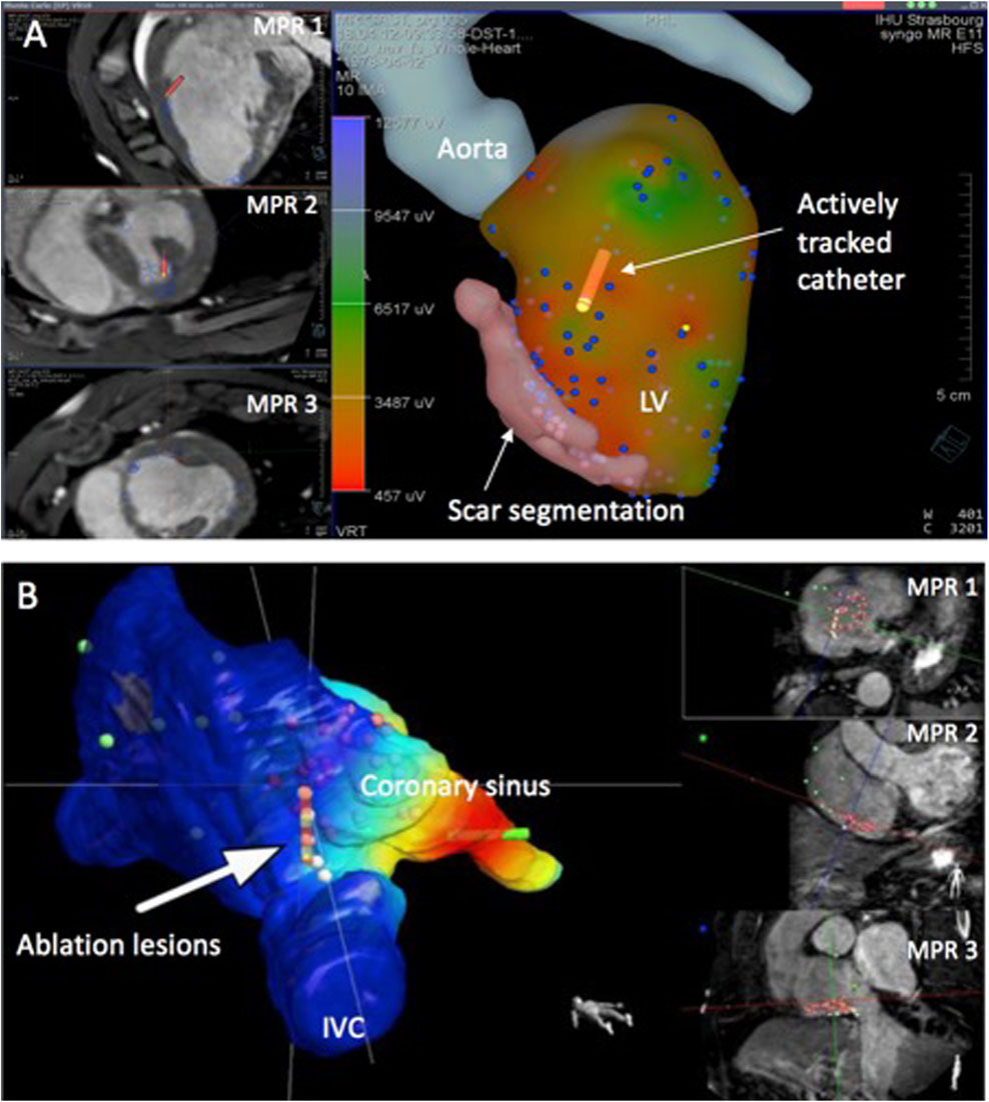
Image guidance software platforms for procedure visualisation have been developed by several MRI vendors that are capable of displaying 3D volume roadmaps, real-time imaging planes, cardiac chamber segmentations, electroanatomical maps and actively tracked catheters. Shown here are example screenshots from the Siemens iCMR platform (**a**) during MRI-guided electroanatomical mapping of the left ventricle in a porcine infarct model and Philips iSuite platform (**b**) during MRI-guided catheter ablation of the cavotricuspid isthmus in a patient with atrial flutter. (Panel **b** adapted from Chubb et al. JACC Clinical Electrophysiology 2017: 3; 89–103, with permission from Elsevier) [20••]. LV left ventricle, IVC inferior vena cava, MPR multi-plane reconstruction

EAM systems are in widespread use for catheter ablation of complex arrhythmias. They can facilitate an understanding of arrhythmia mechanism, permit annotation of single or multiple sites of interest, guide catheter navigation to target ablation sites and, when properly used, can dramatically reduce fluoroscopy times and may reduce procedural times [1]. There has therefore been interest in the development of graphical interfaces to integrate data from real-time imaging into interfaces that closely mimic those of clinical style EAM systems in order to drive progress in real-time MRI-guided electrophysiology [34]. Several major MRI vendors have developed graphical solutions to represent electrical data, which enable real-time imaging with active catheter tracking and workflows for catheter ablation (Fig. 5). Philips Healthcare has developed the iSuite system that consists of hardware including a PC and monitor connected to the MR scanner console. A foot pedal also allows an operator to directly control the scanner in real-time from the scanner bore. Additional software capabilities include the ability to perform MRI-guided navigation combining pre-acquired imaging with real-time data, segmentation tools for procedural planning, as well as custom modules to perform MR-based device tracking and support proton-resonance frequency-based temperature mapping (MR-thermometry) that may have applications to assess catheter ablation [8, 20••].

Similarly, Siemens Healthineers have developed their own interventional MRI platform that allows flexible control of scan plane orientation and image parameters from a dedicated PC connected to the scanner console, load and display volumetric data onto a custom software as well as perform automatic segmentations of pre-acquired data to ensure rapid image processing [32]. The mapping interface of the software gives the ability to change the colour or rendering styles of loaded segmentations and generate activation and voltage maps based on local activation time or voltage amplitude data using colour interpolation between mapping points. Active tracking can be superimposed onto real-time imaging datasets allowing the system to deliver the features required of a clinical EAM system as well as having additional capabilities to exploit the benefits of real-time imaging [32].

The Vurtigo platform, which is compatible with GE systems, is a real-time visualisation application (open-source) that can either import EAMs generated conventionally or compose one using MR-tracked catheters followed by fusion with acquired MR volumes [29]. A real-time scan control system (RTHawk research platform) which is based on the Heart Vista Cardiac operating system (Heart Vista, Los Altos, CA, USA) acts as a 2D viewer and allows acquisition of real-time sequences. The communication latency between the RTHawk application and Vurtigo has been estimated to be in the region of 6.3 ± 7.7 ms [35]. The system has the ability to visualise, compare and overlay MRI volumes, real-time imaging planes, catheters and surface meshes of EAMs. Further developments of features including motion correction of mapping data points and improved signal processing are under evaluation [35].

These systems will require clinical validation to assess the accuracy of the synchronisation and software algorithms to evaluate if image guidance platforms can impact on the safety of electrophysiology procedures through direct monitoring of structures such as the oesophagus, coronary vessels and pericardium during ablation. Further development and refinement of image guidance platforms to display real-time assessment of tissue temperature and predicted tissue necrosis visualise interactions between the catheter tip and endocardial tissue using interleaved cine imaging during active tracking, and compensation for cardiac and respiratory motion during ablation is an exciting prospect. The development of automated mapping systems integrated with image guidance platforms will be a major advance in the field, but these systems and associated algorithms will also require robust clinical validation.

## Ablation Lesion Assessment

MRI offers superior soft tissue visualisation compared to other imaging modalities and has been used to assess acute oedema using T2-weighted imaging and lesion necrosis using late gadolinium enhancement (LGE) after catheter ablation [36]. T2-weighted imaging however is a poor predictor of chronic lesion volume [37]. The use of LGE in acute lesion assessment suffers from variable wash-in and wash-out kinetics of the contrast agent which can lead to the area of necrosis requiring up to an hour to fully enhance [38]. The presence of acute reversible post-ablation oedema may also manifest as increased LGE [39]. Recently, in a canine model, the microvascular obstruction region on LGE images acquired 26 min after contrast administration was found to most accurately predict chronic lesion volume [40] but acute LGE may still grossly overestimate chronic lesion volume by a factor of 3.6 [38]. Contrast can generally only be given once during a real-time MRI-guided procedure and there has therefore been interest in the development of intrinsic (non-contrast)-based methods for ablation lesion assessment [41].

Guttman et al. [42] have described a technique, whereby an inversion recovery T1-weighted gradient-echo sequence with a long inversion time (TI = 700 ms) can be used to visualise lesion necrosis. This approach allowed the discrimination between acute ablation lesion and chronic scar in a porcine infarct model whilst ablation lesion enhancement was less time-dependent and more specific compared to LGE. The time course of lesion visualisation of a T1-weighted inversion recovery balanced steady-state free precession (IR-bSSFP) technique and T2 maps has also been reported with an increase in lesion volume in T2-derived imaging over a 3-h period whilst T1-derived lesion volume remained approximately stable [43]. This approach combining intrinsic contrast–based methods may provide a means to assess lesion composition over time. Non-contrast based techniques can also be repeated multiple times during a procedure and would be potentially advantageous during a real-time MRI-guided procedure.

**Fig. 6:**
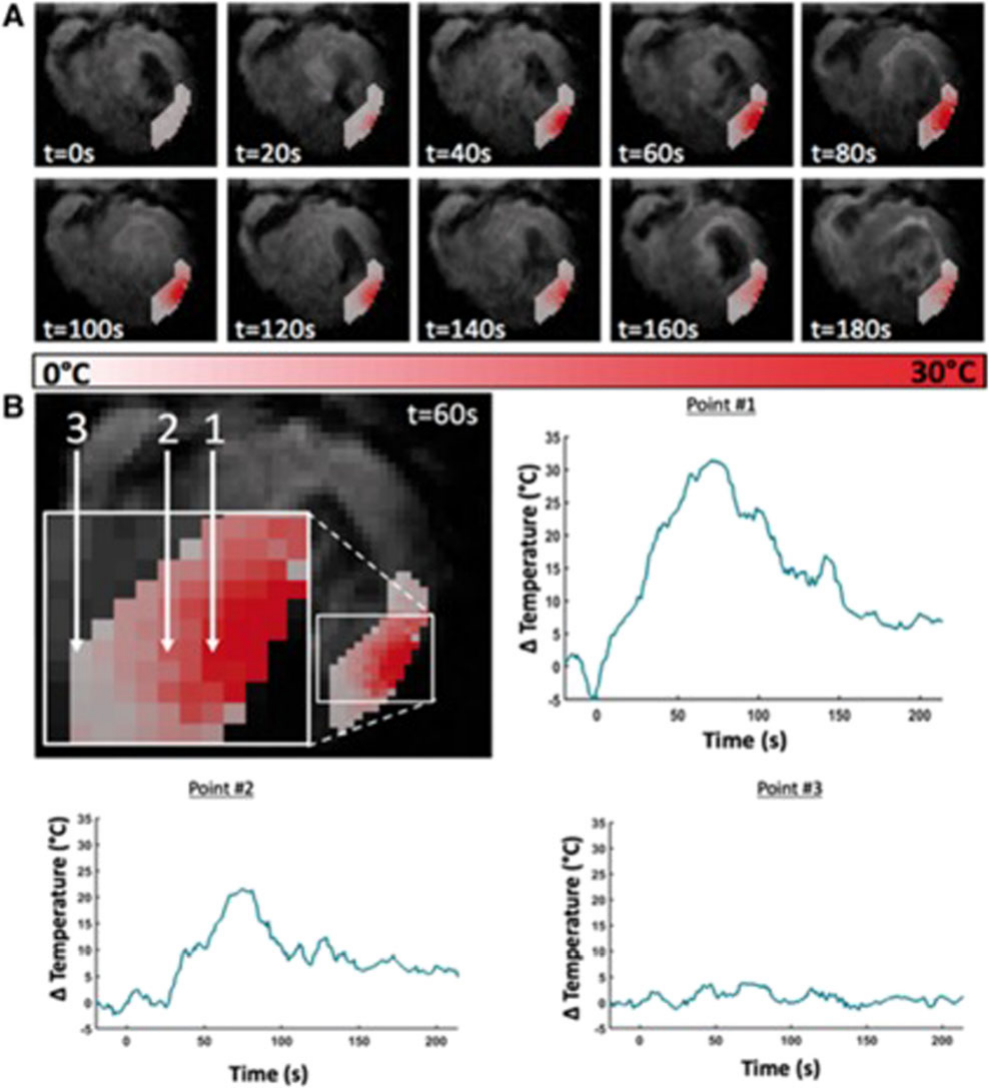
Real-time assessment of ablation lesions using MR-thermometry (**a**) with corresponding temperature profiles (**b**). (Reproduced with permission from Mukherjee et al. Europace 2018 Sep 1;20(FI2):f254-f262. doi: 10.1093/europace/eux341; Creative Commons user license https://creativecommons.org/licenses/by/4.0/) [32].

MRI can also allow the online monitoring of temperature in different tissues as the longitudinal and transversal relaxation time as well as the resonance frequency of water protons are temperature-dependent [44, 45]. The proton resonance frequency shift (PRF) technique [46] exploits the latter property to generate information on temperature during a gradient echo pulse sequence in each pixel of an image (MR-thermometry–Fig. 6). The thermometry technique can be combined with a calculation of thermal dose (MR-dosimetry) whereby the integral of temperature elevation and time is computed to derive a dose that predicts when destruction of the target tissue is achieved [44, 47]. When applied in a moving organ such as in the heart, physiological motion and its associated thermometry artefacts can lead to important errors that need to be corrected [44, 48, 49•,^50^–^52^].

MR-thermometry and dosimetry could potentially allow a precise means of assessing the efficacy of ablation during real-time MRI-guided electrophysiology and has been studied in pre-clinical models. A multi-slice, ECG-triggered, MR-thermometry pipeline has been used in an ovine model to demonstrate a temperature increase of up to 38° near the catheter tip during RF ablation [48]. Further, for the assessment of endocardial acute ablation lesions, lesion dimensions on thermal dose maps derived from MR-thermometry correlated to dimensions acquired using a T1-weighted gradient echo sequence and gross pathological examination [49•]. This technique has been extended to epicardial ablation in a porcine model whereby lesion dimensions on thermal dose maps within a precision of 1 mm of those measured post-mortem was achieved [32] similar to the precision of MR-dosimetry of endocardial ablation reported by the Bordeaux group [49•]. Several obstacles remain to be overcome for the thermometry technique including accurate motion estimation due to cardiac and respiratory motion, intra-scan motion, and inter-scan motion. The presence of cardiac arrhythmias can also lead to difficulties during image acquisition which is ECG-triggered. It remains unclear whether thermal dose achieved during the real-time procedure can predict the durability of chronic lesions [32].

## Real-Time MR-Guided Electrophysiology for Atrial Arrhythmias

The demonstration of feasibility of real-time MRI-guided electrophysiology in patients has largely focused on the atrium, in part due to ease of access and safety. Diagnostic and ablation studies have been completed in the right atrium of pre-clinical models using steerable, non-ferromagnetic MR-compatible catheters [53, 54] including at 3T [55]. Clinical translation of the technology has also been achieved in patients with atrial flutter using passive tracking [24, 56•] and active catheter tracking [19••, 20••].

Atrial flutter ablation using conventional electrophysiology systems however has excellent long-term clinical outcomes, a low risk of complications [57] and is unlikely to be improved meaningfully by real-time MRI guidance. Typical atrial flutter is a very well understood arrhythmia with a proven interventional treatment and has been an excellent testing ground in which to develop clinical workflows and demonstrate feasibility of the technology. There is interest, however, in the development of catheters and technologies to perform complex arrhythmia ablation inside the MRI scanner including for atrial fibrillation (AF). Using active tracking, MR-compatible catheters have been navigated into the left atrium via a trans-septal puncture to perform left atrial mapping and pulmonary vein circumferential ablation [58]. A major limitation for real-time MRI-guided electrophysiology has been the availability of devices that can be used inside the MRI scanner. Almost all real-time MRI-guided studies to date have used single bipole (tip and ring electrode) catheters using point-by-point mapping. Recently, the feasibility and safety of a multi-electrode (10-poles) MR-compatible circular mapping catheter was evaluated in an ovine model [21•]. A MRI-based cryoablation system has also been developed and used in canines to perform circumferential ablation of the superior vena cava-right atrial (SVC-RA) junction with no gaps [59•] and in the pulmonary veins (PVs) with 95% of the PV ostia covered with scar at 3 months [60]. Availability of MR-compatible trans-septal puncture kits will also be a pre-requisite for MR-guided AF ablation. An active MR intra-vascular needle system has been used in a porcine model to safely puncture the fossa ovalis via a transfemoral approach whilst allowing direct visualisation of the needle, atria and surrounding vasculature [61] but clinical validation of these devices are currently lacking. Further development and investment in these devices through industrial collaboration are likely to accelerate progress in the application of real-time MR-guided electrophysiology for the treatment of AF.

Another potential application of real-time MRI guidance could be in patients with congenital heart disease (CHD) requiring catheter ablation for atrial tachycardias. Although interventional MRI for cardiovascular applications was initially developed in children with CHD to enable cardiac catheterisation without the use of fluoroscopy and limit radiation exposure [62], there has been no work on the application of real-time MRI to guide electrophysiology procedures in these groups of patients. Arrhythmias are a common cause of morbidity and mortality in these patients, and there may be benefits of accurate anatomical evaluation and substrate assessment with MRI in CHD patients with scar-related atrial tachycardias. Future studies in this cohort are anticipated.

## Real-Time MR-Guided Electrophysiology for Ventricular Arrhythmias

The application of real-time MRI guidance for electrophysiology procedures in the ventricle is likely to be the area where MR-based solutions may have their greatest impact. Catheter ablation for scar-related ventricular tachycardia (VT) is associated with modest success rates and a high rate of complications including early mortality [63]. The use of real-time MRI guidance for 3D substrate assessment at the time of procedure, accurate guidance of catheters to target regions of interest and utilising real-time techniques to evaluate ablation lesions (e.g. MR-thermometry) could theoretically improve the efficacy and safety of VT ablation [12]. There are numerous challenges however prior to the realisation of VT ablation under real-time MRI guidance. The current generation of MR-compatible catheters may require modifications to improve manoeuvrability, torque transfer and handling capabilities to enable mapping and ablation via retrograde, trans-septal and epicardial access to the LV. The inability to perform defibrillation safely inside a MRI scanner represents a major hurdle and is a pre-requisite for clinical studies in patients. To date, there are only limited reports of prototype MRI-conditional defibrillation systems to enable electrical defibrillation both inside and outside the scanner bore in swine [64•]. Routine availability of high-fidelity 12-lead ECG systems for use inside the MRI scanner is also limited [16] and will be valuable for diagnostic and monitoring purposes during VT ablation. The majority of patients who undergo VT ablation have an implantable cardioverter defibrillator (ICD) in situ [65]. Although MRI has been shown to be safe for imaging patients with devices, including legacy ICD systems [66], there are concerns regarding radiofrequency-induced tissue heating and device failure and their potential impact during long procedures lasting several hours, as is often the case during VT ablation. The presence of ICDs also leads to the generation of artefacts; although wideband LGE sequences have been reported to adequately suppress artefacts and preserve diagnostic image quality for LGE imaging [67, 68], this remains a challenge for real-time MRI-guided procedures.

Real-time MRI-guided electrophysiology studies have been performed in animal models of myocardial infarction to measure EGMs within scar and generate voltage maps in the LV using both retrograde and trans-septal approaches [69]. In a separate study, MR-derived borderzone tissue exhibited more abnormal potentials than dense scar or healthy myocardium in a porcine infarct model using a real-time MRI-guided system where there was no requirement for registration between electroanatomical maps and MRI-derived substrate [22]. Real-time systems have also been used to perform epicardial mapping and ablation in the normal swine left ventricle [32]. There are no studies to date evaluating real-time MRI-guided systems in the ventricle of patients with cardiac arrhythmias. Due to the limitations noted above, clinical translation to VT ablation will be difficult to achieve in the next few years. As an intermediate step, catheter ablation under real-time MRI guidance in patients with idiopathic premature ventricular contractions (PVCs) may be more feasible. PVC ablation either in the RV or LV outflow tract with direct assessment of lesion formation using real-time MRI techniques could lead to further technological developments to realise the ultimate goal of MRI-guided VT ablation in patients with ischaemic heart disease.

## Conclusions

There is significant potential for real-time MRI-guided electrophysiology to transform intervention for cardiac arrhythmias but challenges remain. In order for real-time MRI-guided procedures to become viable, an increased availability and range of MR-compatible devices is essential and will require close collaboration between traditionally distinct clinical groups (interventional electrophysiology and MR radiology), academic institutions and industry. Routine adoption of the technology will only gain momentum with the objective demonstration of clinical benefit for patients at a reasonable cost.
